# Germline whole genome sequencing in pediatric oncology in Denmark—Practitioner perspectives

**DOI:** 10.1002/mgg3.1276

**Published:** 2020-06-04

**Authors:** Anna Byrjalsen, Ulrik K. Stoltze, Anders Castor, Ayo Wahlberg

**Affiliations:** ^1^ Department of Clinical Genetics Copenhagen University Hospital Rigshospitalet Copenhagen Denmark; ^2^ Department of Pediatric and Adolescent Medicine Copenhagen University Hospital Rigshospitalet Copenhagen Denmark; ^3^ Department of Paediatrics Skaane University Hospital Lund Sweden; ^4^ Department of Anthropology University of Copenhagen Copenhagen Denmark

**Keywords:** clinical genetics, ethics, pediatric oncology, practitioner perspectives, whole genome sequencing

## Abstract

**Background:**

With the implementation of a research project providing whole genome sequencing (WGS) to all pediatric cancer patients in Denmark (2016–2019), we sought to investigate healthcare professionals' views on WGS as it was actively being implemented in pediatric oncology.

**Methods:**

Semistructured interviews were carried out with pediatric oncologists, clinical geneticists, and research coordinating nurses (*N* = 17), followed by content analysis of transcribed interviews. Interviews were supplemented by ethnographic observations on Danish pediatric oncology wards. Additionally, questionnaires were distributed to healthcare professionals concerning when they found it appropriate to approach families regarding WGS. The response rate was 74%.

**Results:**

Healthcare professionals see imbalances in doctor–patient relationship, especially the double role doctors have as clinicians and researchers. Some were concerned that it might not be possible to obtain meaningful informed consent from all families following diagnosis. Still, 94% of respondents found it acceptable to approach families during the first 4 weeks from the child's diagnosis. Views on the utility of WGS, treatment adaptation, and surveillance differed among interviewees.

**Conclusion:**

Overall, healthcare professionals see dilemmas arising from WGS in the pediatric oncology clinic, and some advocate for further educational sessions with families and healthcare professionals. Despite concerns, healthcare professionals overwhelmingly supported early approach of families regarding WGS. Interviewees disagree on the benefits of surveillance based on genetic findings.

## Graphical abstract

Healthcare professionals see dilemmas arising from whole genome sequencing (WGS) in the pediatric oncology clinic, and some advocate for further educational sessions with families and healthcare professionals. Despite concerns, healthcare professionals overwhelmingly supported early approach of families regarding WGS. Interviewees disagree on the benefits of surveillance based on genetic findings.

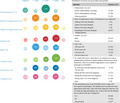

## 1 INTRODUCTION

Pediatric oncology is a research‐intensive medical specialty in Denmark, as it is globally. Societal attention, ear‐marked research funds, and specialized research laboratories have contributed to the establishment of extensive research programs across Denmark's four pediatric oncology centers. And so, while medical practitioners on pediatric oncology wards are focused on providing the best possible treatment and care for their patients, the integration of research within the clinic shapes daily treatment routines and patient treatment trajectories. Oncologists are tasked with introducing families to various ongoing research projects, and clinic nurses are asked to help collect samples, forms, and questionnaires for these projects. Such efforts must coexist with their primary responsibility; caring for their patients, and finding the right balance can be a challenge especially when some patients are eligible for five to 10 projects at the time of inclusion.

Indeed, clinical care and research are now so intertwined that Cambrosio and colleagues have argued that a longstanding tradition for translational research has contributed to what they see as “oncology's fading boundary between research and care” (Cambrosio, Keating, Vignola‐Gagné, Besle, & Bourret, 2018).

They argue that the field of genomics has accelerated within oncological research, fueled by “personalized medicine” research agendas and falling sequencing costs. As a direct consequence, a string of qualitative studies has in recent years investigated medical practitioner perspectives on whole genome sequencing (WGS; Christensen et al., 2016; Lemke, Bick, Dimmock, Simpson, & Veith, 2013; Lohn, Adam, Birch, Townsend, & Friedman, 2013; Vassy et al., 2015). While each of these studies have prospectively asked practitioners about their views at a time when “WGS may soon play an important role in primary and specialty care” (Christensen et al., 2016), our study is different. We have investigated practitioner perspectives on WGS at a time when it is being actively integrated within an entire nationwide specialty, namely pediatric oncology in Denmark, through the Sequencing Tumor and Germline DNA—Implications and National Guidelines (STAGING) project, providing WGS to all newly diagnosed pediatric cancer patients in Denmark. STAGING maps frequency of cancer predisposition syndromes in a consecutive, national cohort of children with cancer. This knowledge is used for treatment adaptation, implementing relevant surveillance measures and family planning. The present study was designed and carried out by an anthropologist (AW) and a clinical geneticist (AB) to investigate *how pediatric oncologists, clinical geneticists and nurses perceived, reflected on and reacted to the introduction of WGS in the clinic in real time through a research project and when they found it appropriate to approach families about WGS research*. As we will show in what follows, this introduction has not been without friction, raising both ethical and practical dilemmas, yet it is feasible to implement, and healthcare professionals are overall supportive of WGS research (Byrjalsen et al., 2018; Johnson et al., 2017).

## 2 MATERIAL AND METHODS

The qualitative part of the present study is based on semistructured interviews carried out with 12 pediatric oncologists, three clinical geneticists, and two research coordinator nurses by the anthropologist (*N* = 17). AB has been responsible for recruiting over 240 families into the STAGING project and has had direct interactions with treating physicians, nurses, laboratory technicians, and research coordinators over the last years (2016–2019). ABs direct experiences of recruiting for STAGING and AWs participation in various debates and discussions at seminars form an important backdrop to the interview data.

All interviewed healthcare practitioners have played a role in STAGING, whether as treating physician or directly involved in STAGING (recruitment, genetic counseling, or sample collection). Interviews lasted between 45 and 90 min and took place at a time and place convenient for interviewees. The semistructured interviews were based on an interview guide (Box 1) covering the topics of the importance of research in pediatric oncology, introduction of WGS to families at the time of diagnosis as well as perceived challenges and dilemmas. All interviews were recorded and transcribed verbatim by research assistants with quality control by AW.

BOX 1 **Questions semi‐structured interview guide**

- Why is it important to carry out as much research as you do on the pediatric oncology ward?
- What do you need to be mindful of when you approach families about participation in a research project?
- What do you see as some of the specific or important ethical issues that whole genome sequencing gives rise to for families?
- In which ways do you envisage genetic information will be relevant for you as a practitioner?

Our questionnaire was developed by AB and AW based on concerns raised in interviews with parents (Byrjalsen et al., 2018) and healthcare professionals, to assess when it would be appropriate according to healthcare professionals to approach families about WGS. Prior to distribution, the questionnaire was revised based on comments by a sociologist and other members of the STAGING research team. The questionnaire was distributed via e‐mail to 73 employees employed at either Rigshospitalet's Department of Pediatric Oncology or at one of the Clinical Genetics Departments in Denmark. Invited employees included 45 nurses, 15 doctors, and 13 clinical geneticists, including employees involved in STAGING. In addition to an oral reminder from senior pediatric oncologists, reminders were distributed via e‐mail three times after 4, 8 and 12 weeks from distribution. Of the 73 employees who received the questionnaire, 54 employees participated rendering a participation rate of 74%. Nineteen (26%) did not participate in the study. All 15 (100.0%) pediatric oncologists, 27 of 45 (60.0%) nurses, and 12 of the 13 (92.3%) clinical geneticists participated (Table 1).

**TABLE 1:** Questionnaire distributed to healthcare professionals. Distribution of answers are given under each question

Question	Answer, *n* (%)
1. Employment: Are you?	
Doctor within pediatric oncology	15 (28)
Nurse within pediatric oncology	27 (50)
Clinical geneticist	12 (22)
2. Place of employment: where in Denmark are you employed?	
Aalborg University Hospital	1 (2)
Aarhus University Hospital	3 (6)
Odense University Hospital	3 (6)
Copenhagen University Hospital (Rigshospitalet)	46 (87)
3. Gender: Are you?	
Male	10 (19)
Female	43 (81)
4. Age: I am…	
<40 years old	22 (42)
40–50 years old	16 (30)
>50 years old	13 (25)
I do not wish to say	2 (4)
5. In a child with cancer, where there is no suspicion of a cancer predisposition syndrome or family pedigree with massive cancer disposition, when do you think it appropriate to ask a family to participate in STAGING?	
In connection with the diagnostic interviewor the day after	5 (10)
During the first week from diagnosis	12 (23)
During the second week from diagnosis	11 (21)
During week 3 or 4 from diagnosis	21 (40)
Later	3 (6)
6. The participation rate in the STAGING study is high (>90 have agreed to participate). The families have not until now been approached earlier than 2 days after their diagnostic interview. Do you think the high participation rate, is due to the fact that families are not asked on the day of diagnosis?	
Yes	26 (50)
No	13 (25)
I do not know	13 (25)

We carried out content analysis of the interview data generated (Hsieh & Shannon, 2005) with both anthropologist and clinical geneticist reading each interview transcript carefully focusing on how informants view the *introduction of* WGS to families, the boundary between treatment/care and research, how to obtain informed consent and potential consequences of WGS. It has not been our intention to compare views across subgroups rather we have treated our informant group as a whole, that is, those healthcare professionals who have played a role in the introduction of WGS into pediatric oncology in Denmark.

Transcripts were read twice to ensure full data immersion, with a series of three coding meetings between AB and AW to discuss and agree on identified themes. Meetings were also used to relate the views and response patterns found in the interview transcripts to our own experiences in the clinic and in our interactions with clinicians (Spradley, 2016).

Study questionnaire responses were aggregated and divided based on gender, educational background, and age. We initially performed Chi‐squared tests in order to identify potential differences between groups, none of the analysis reached statistical significance, likely due to the small sample size, and we opted not to include these results. The questionnaire study was supplemental to our overall aim of exploring healthcare professionals' views on the introduction of WGS into pediatric oncology.

## 3 RESULTS

### 3.1 Doctor, researcher, or both?

Whole genome sequencing has been introduced into pediatric oncology in Denmark through the STAGING research project. All interviewees insisted that research is fundamental to the treatment of children with cancer, with a few suggesting that *not* gaining as much knowledge as possible from every single patient was immoral. At the same time, research within pediatric oncology healthcare inevitably raises ethical challenges for professionals due to imbalances in doctor–patient relationship. For families in crisis in a welfare state like Denmark, trust in authority and dependence prevails, particularly as regards their primary treating physician.

So when you go into a family's ward room, where you are both telling them about their child's diagnosis and in the same breath introducing them to research, you have to be very careful in choosing your words. You have to consider very carefully what kind of power imbalance you are in the midst of (Pediatric Oncologist B)

This imbalance can also reveal itself through what is actually said to families as physicians maintain a kind of “editorial power” when it comes to dispensing information. Many doctors face an embedded conflict as they oftentimes have stakes in both the treatment provided and research conducted:

At times I think I may ‘oversell’ research projects. Especially projects that I am an active part of. … Afterwards I’ll reflect on whether my presentation was fair or not and there are times where I've thought ‘that was borderline’, but I haven't thought it was wrong… You do [as a doctor] have enormous power… At times I have thought that I've stood there explaining something where they… They say ‘yes’, but actually they don't know what they are saying ‘yes’ to. And I don't feel so good about that. I mean… I'm not trying to manipulate them into saying ‘yes’, because for me it is definitely legitimate to say ‘no’ … On the other hand, some families might end up saying 'no' to something where I'm thinking "this would have been a really good opportunity for you"… So, there are times when we are left with a bitter taste in our mouths, wondering whether we have done the right thing (Pediatric Oncologist C)

As described here, the lines between physician and researcher roles can get blurred, which raises questions about the possibility of undue influence, whereby the doctor's own enthusiasm for a project risks swaying patients and their families' views (Dekking et al., 2015). Almost all interviewed pediatric oncologists agreed that this is an unavoidable consequence of the doctor–patient relationship, but also in any human relation:

I don't think we can escape this issue. You can try to reduce the consequences by being aware, humble and open about it. You could get someone not related to any part of the research project to provide information about the project, but then again they wouldn't be able to explain it in sufficient detail. And, as soon as you have gained sufficient knowledge you are a part of it (Pediatric Oncologist B)

When asked about undue influence another doctor mulled over the idea of being two doctors informing families about research together, which he surmised might make both more conscious about the way they choose their words. He continued:

…we have implemented this study because we want patients to participate. I think it's okay to say ‘we hope you will consider it’ and that you want them to participate. But you can't pressure them to do so (Pediatric Oncologist D)

A project coordinating nurse noted that influence was indeed at times used as a way to move things along.

When I have followed up with a family 3 or 4 times, I might ask them if they have any further questions. And sometimes I namedrop a certain doctor, and explain that this is his project, and that they can also ask him. It's a bit subconscious I think, because I know they like him (Research Coordinator A)

A final theme concerning doctor–patient/family relationship in research recruitment that emerged out of our interviews was that of paternalism, and there seemed to be a shared sense that a degree of paternalism is inevitable.

We [pediatric oncologists] have always thought in terms of *our* patients. It may well be because we fight so much by their side. But in all honesty, they are not ours, they are their own… So, there is a balance there, in that we should not become too paternalistic towards them (Pediatric Oncologist E)

This doctor went on to describe an episode concerning a family that had requested more time before being presented with a specific project. The patient suddenly deteriorated, and the researchers reached out to the doctor asking her to talk to the family again as securing biological material at this stage would allow the family to participate later if the patient did not make it. This would be crossing a line for the doctor:

This is the only patient where I have said ‘I can't do this. This is unethical’. They have had a window at a less critical time. They have already been given the opportunity… But I have also wondered whether this was the right decision (Pediatric Oncologist E)

### 3.2 Meaningful informed consent to WGS research

Related to the question of imbalances in relations between healthcare professionals and their patients, is the question of meaningful informed consent (Oberg et al., 2015). During the first weeks on an oncology ward, families must digest complex information about diagnostics, treatment plans, potential side effects, and prognoses, while additionally being introduced to five to 10 different research projects (Byrjalsen et al., 2018). Given this overload of information, communication is to a large extent “based on trust.” However, pediatric oncologists are not always convinced that families have fully understood what has been asked of them:

I do worry that there are people who would say ‘yes’ to something that they don't understand. I mean, if I had just found out that my child will die or has a terminal diagnosis… I mean that's what many people are thinking when they are told “your child has cancer”, they're thinking “my child is going to die”. You shut things out at that point, I mean I think your brain shuts down… You can't imagine anything worse. Going from there to giving informed consent to something as complex, well I guess I do have my doubts as to whether they are in a position to take it all in… (Pediatric Oncologist N)

Oncologists are also cognizant of how families have come through the hospital doors. Some families have literally gone to the doctor concerned about a flu only to find out that their child has cancer on the same day, while others have been trying for months to find answers to explain their child's symptoms before finally receiving the cancer diagnosis. Doctors try to navigate how much information a family can digest at any given time:

…I do sometimes introduce research projects on day one… But then there are other families where you can see that they are so affected that they won't have understood anything I've said even before they came here… I mean, they are not in a place where they can understand anything. And it is difficult then to inform them about any of our research projects. We ask them to consider very complicated things, really already on day 1, where it is probably almost unethical sometimes to ask them to consider such things. We're pretty much speaking in two different languages at that point … And that is regardless of a families' educational background. I mean for me there is no difference whether I am presenting a research project to someone from social class 4 or social class 1 (Pediatric Oncologist C)

While these factors are relevant for all research, some pediatric oncologists pointed out that they thought that WGS presented specific challenges because of the complexities involved. Before parents' consent to STAGING, they go through genetic counseling during which potential consequences of WGS including the risk of secondary findings are raised. Some practitioners were, however, unsure whether families are able to sufficiently grasp the potential consequences:

I mean, I do think that the challenge with WGS is that it just is so difficult to grasp. It is true both for us, but perhaps even more so for your average person … say you were to find out that your child has a *BRCA2* gene defect and will have an 80% risk of getting breast cancer or ovarian cancer. First, what if you find this out prenatally, should one say ‘no thanks’ to having a child with that gene defect? If they are born, should one offer them a mastectomy? When? When should we remove their ovaries? When is the right time and when is it too late? I mean, you know. And we as doctors, me – who tends to think mathematically – I find it very difficult. And that's what then leads me to ask whether it is at all possible to give fully informed consent (Pediatric Oncologist D)

**FIGURE 1:**
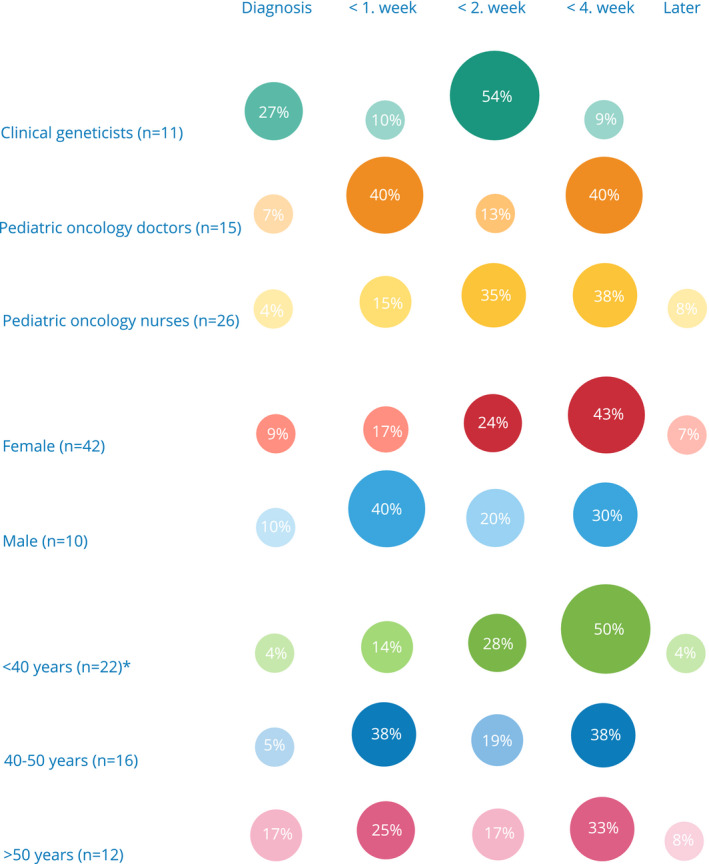
Healthcare professional's opinion of what constitutes the appropriate time from diagnosis to approach about STAGING as distributed according to job title, gender, and age. *Two respondents did not fill out the age range question

Against this backdrop of practitioner reflections about difficulties in obtaining meaningful informed consent to WGS, in our questionnaire study 10% of employees found it appropriate to approach families about germline WGS at the time of diagnosis, half of all respondents found it appropriate during the first 2 weeks (23% during the first week and 21% during the second week), 40% found it appropriate during the first 4 weeks from diagnosis, and 6% thought it should be scheduled after 1 month from diagnosis (Table 1). Clinical geneticists opted for either introduction at diagnosis or during the first week (37%) or during week 4 or after (63%). For pediatric oncologists these numbers were 47% at diagnosis or during the first week, 13% during the second week from diagnosis and 40% during the first 4 weeks. No one opted for approach after the first month. Among nurses, 19% opted for approach at diagnosis and during the first week, 35% opted for approach during the second week, and 46% opted for approach during week 4 or later (Figure 1). When subdividing according to gender, there was a tendency toward male employees preferring an earlier approach than women (there were no males among nurses). Although 10% of participating women and men found approach at diagnosis appropriate, 40% of males found it appropriate during the first week, compared to only 16.6% of women. Conversely, 74% of women thought it appropriate during or after the second week following diagnosis, compared to 50% of men (Figure 1).

A final theme to emerge around the obtaining of meaningful informed consent to WGS was that the introduction of WGS into the pediatric oncology clinic in Denmark is not only new for families, it is also new for pediatric oncologists, some of whom do not consider themselves sufficiently equipped to answer all potential questions from families. This raised concerns about how an introduction of WGS into the clinic can be professionally supported:

Well, I would say that the first challenge is that I am from exactly that generation which didn't have much exposure to genetics, right? I'm also from that generation who didn't have a computer in school. The last generation! So many of us doctors who are around my age, who work here, who are in their 40s or 50s, we don't have much knowledge about it. At least not the technical knowledge and things like how sure we are about the findings, how uncertain the numbers are, etc. So, we lack specialized education, you could say, we lack knowledge about it (Pediatric Oncologist D)

### 3.3 Potential consequences and utility

Genetic counseling and testing have traditionally been performed weeks or months after a serious diagnosis. More recently, as in the case of STAGING, counseling and testing have moved closer to the time of diagnosis to allow for treatment options based on genetic predisposition. And while the clinical geneticists interviewed for this study were not specifically asked about this development, it often emerged. Some were nervous that families would agree to something out of fear of losing their child, others did not see the need for burdening families at such an “early stage.” Still others argued that minimizing information prior to genetic testing might be beneficial, and that testing should be a clinical decision and that doctors should provide in depth counseling only when results had revealed a predisposition syndrome.

Guilt is omnipresent within genetics both as regards the choice to undergo genetic testing or not and in terms of “survivor's guilt,” where family members not carrying the family's pathogenic variant feel guilt over their “luck” (Lerman & Croyle, 1996). As one clinical geneticist in the study put it *parents always feel guilty if they have passed “bad” genes on to their children*. Yet, one of the pediatric oncologists we interviewed insisted that the issue of guilt is not specific to genetics:

We may well risk taking families hostage in our search for answers [about cancer etiology] and guilt is a big part of that… But guilt is not confined to WGS. Choosing to enroll in a randomized treatment trial harbors the same risk of guilt, because if the child relapses, parents will think ‘is this my fault? I chose to enroll my child in this trial’ (Pediatric Oncologist B)

Another issue regarding WGS is that other family members risk gaining information that they have not asked for:

When you get knowledge of a genetic mutation in the family you are forced to think in family relations. That is new to many patients. They often have contact with close relatives but not necessarily those further out in their family. That puts it into a whole other perspective; people you share genes and diseases with are sometimes strangers (Clinical Geneticist J)

However, genetic testing may also give some families reassurance regarding thoughts they are already burdened by:

I don't think a couple who have had a child with cancer has ever thought ‘this will never return’ or ‘now we are home safe’. But in this case, we will be able to say that ‘we have done everything we could and there is no increased risk’ whereas in the case of any findings we will be able to say ‘there is something genetic in your child's DNA and now we can watch out for the cancers there is a greater risk of developing’. I think we take better care of these families through this (Clinical Geneticist K)

When doing WGS there is a risk of running into secondary findings; that is genetic findings that were not of primary interest, but are none the less identified and assessed. However—as pointed out by one clinical geneticist—this is always a risk when patients come into contact with the healthcare system:

Every time someone contacts a doctor they may end up diagnosed with something they didn't expect. We may be looking for pneumonia but end up finding lung cancer… The important thing is that we as medical professionals’ follow‐up when something pathogenic turns up (Clinical Geneticist K)

Healthcare professionals' views regarding families' abilities to cope with genetic information differed. Some thought families would have difficulties understanding it, while others did not share that concern. One pediatric oncologist argued:

They do risk being taken hostage, ending up with some information they didn't need to have. They may opt out of receiving those, but there are some findings that they cannot opt out of, so they receive those findings regardless. And that is taking them hostage to some degree… but such hostage taking is present in so much of the clinical and research work we do (Pediatric Oncologist B)

If a genetic predisposition is found, enrollment in a relevant clinical surveillance program may be a consequence. Surveillance programs within pediatric oncology are, however, debated:

There could be some surveillance that would be different [for a child with a germline predisposition] to what we do now. Or for other family members. And it may be significant. But we need to be critical, we should not do all sorts of tests without knowing that it can benefit the patient… you risk stigmatization (Pediatric Oncologist L)

There are screening programs that are well documented and can increase survival and decrease toxicities, but we don't have a lot of these diseases yet… I am against screening, but I think we need to be hesitant regarding screenings that don't make a difference (Pediatric Oncologist F)

A specific point of disagreement involved how to handle patients diagnosed with Li Fraumeni syndrome, resulting in a significant lifetime risk of developing various and multiple primary cancers. Studies of the so‐called Toronto protocol (Villani et al., 2016) suggest that comprehensive surveillance efforts are beneficial yet these studies have been highly debated within the community. Some pediatric oncologists touched upon this:

For the patients with a *TP53* mutation we've had discussions. Some think we should initiate excessive surveillance programs, which in my opinion is without scientific evidence in terms of reducing morbidity and mortality (Pediatric Oncologist L)

For Li‐Fraumeni patients, they have an increased risk of cancer, but we don't know which cancer they will develop. And you don't know whether it will occur and where. And that means that either you have to sleep in an MRI scanner for the rest of your life, or then you can live your life and be conscious of symptoms and act accordingly if they should arise (Pediatric Oncologist F)

A competing argument was put forward by both clinical geneticists and pediatric oncologists. As one clinical geneticist put it:

Over time we have found that genetic testing needs to be put up‐front. That it is simply stupid to wait for the second tumor before you think that something may be wrong… You cannot offer a child the best standard of care if you don't know what is waiting around the corner. It would be stupid to give irradiation therapy to a child who has a mutation that results in lower sensitivity to irradiation therapy and an increased risk of a secondary cancer … the more we know the better we are able to help (Clinical Geneticist K)

## 4 DISCUSSION

Through this combined qualitative and quantitative study we have explored the double role held by many healthcare professionals within pediatric oncology, that is, working both in the clinic and in research. While this has been the case for many years by now, we have focused on how this double role relates to the introduction of WGS into pediatric oncology. Primary concerns raised by healthcare professionals regards the potential of undue influence, obtaining meaningful informed consent, and the clinical utility of WGS. These concerns notwithstanding, 94% of respondents found it acceptable to approach families about WGS with the first month from the child's diagnosis. In the following, we will discuss our findings in light of previous research as well as highlight strengths and weaknesses of this study.

### 4.1 Influence—Undue?

Numerous dilemmas concerning autonomy, power imbalances, and consent rise out of the dependency in the doctor–patient relationships. These issues have been described by other previously (De Vries et al., 2011), but with the introduction of WGS into the routine diagnostic care novel complexities arise. All pediatric oncologists interviewed for our study were acutely aware of this and reflected on the responsibilities this relationship engendered. At the same time there is an abiding sense among pediatric oncologists/clinical geneticists that research is completely fundamental to the medical care that is delivered, and an inherent sense of obligation to gain as much knowledge from every case of childhood cancer as possible to better the chance of survival and decrease morbidity for future patients. Our findings are supported by Dekking et al. who also found that “when informing families about research, [pediatric oncologists] sometimes felt a tension between motivating and being too persuasive” and that “research is considered a fundamental and indispensable characteristic of practice” (Dekking et al., 2015). What these findings suggest is that there is a fading boundary between research and care within oncology (Cambrosio et al., 2018). And this calls for active engagement in the clinic with “situated ethics” (Ong & Chen, 2010) whereby healthcare practitioners reflect on and share those cases where, as one pediatric oncologist put it, “we are left with a bitter taste in our mouths, wondering whether we have done the right thing.” Distinguishing influence from undue influence, inducement, or coercion requires dialogue and reflection on the part of practitioners.

One pediatric oncologist pointed out that it could be beneficial to see how communication may change if two colleagues informed families of research alongside each other, arguing that the presentation may be more measured if a colleague were present. This highlights an inherent tension; that doctors who have a personal stake in patients accepting participation in their study are also the people informing them thereof. Addressing this, another pediatric oncologist suggested that you have people not involved with the study inform patients and families about research projects. The problem, however, is that as soon as someone gets a certain level of insight into a project they would have a stake in it. And while it may be technically possible to get qualified candidates to include patients in the study it would be costly and difficult to implement as many biomedical research projects are complicated and require the person informing patients about the study to be educated within the biomedical field, to be able to give adequate information. While there is nothing new about such tension in medical research in the clinic, the introduction of WGS into pediatric oncology through research raises a number of dilemmas that are specific.

### 4.2 Meaningful informed consent to WGS

Informed consent is complex even in the best of circumstances, and as pointed out by numerous interviewees in the study, particularly complex when regarding WGS. Some pediatric oncologists had experienced that families had misunderstood elements when discussing the study after the genetic counseling session. This is a well‐known issue within medicine and clinical genetics—that patients do not recall information given during a clinical or research consultation (Rona et al., 1994). Numerous interviewed pediatric oncologists pointed out that their own understanding of WGS is sparse making it difficult for them to help if patients had further questions. Scollon et al. also found that the communication provided by pediatric oncologists and clinical geneticists when returning results from exome sequencing were largely one‐way communication lacked involvement of parents and patients (Scollon et al., 2019). To improve understanding and recollection numerous suggestions have been made including having two inclusion sessions as a so called “two‐tier model” (Oberg et al., 2015), educational sessions, follow‐up interviews, or questionnaires after the inclusion session.

Other suggestions could be to have patients (in this case parents) go through educational training to ensure they obtained the relevant knowledge. In drug trials it is good clinical practice to have patients repeat what they have been told by the informing doctor, in order to ensure that they have the level of knowledge that is required to handle taking the medication, being aware of side effects, etc. However, further educational sessions at a time when the patient is being treated for cancer, might be infeasible for families given their circumstances. Another option would be to do more general Q&A sessions for participating families allowing for further knowledge to be obtained. The benefit of implementing further educational training is to help participating patients and families be better prepared for eventualities. However, such requirements might risk families opting out, especially families from less‐resourced social backgrounds. As a more feasible option, optional Q&A or educational sessions may be worth exploring to heighten the understanding of the complex information about risk, surveillance, and secondary findings for both patients, families, and healthcare professionals. On this note, it is worth mentioning that information regarding WGS includes information about secondary findings, which by their very nature are impossible to give an exhaustive presentation of.

These findings stand somewhat in contrast to the findings from our questionnaire study in which healthcare professionals overwhelmingly supported introduction to WGS research within the first 4 weeks after diagnosis. This might be because—as argued by one interviewee—the introduction process works “exactly because” the treating physician is asked whether they deem a family ready for approach. Further educational interventions aimed at families do not seem to necessarily hinder approach during the first 4 weeks following diagnosis, indicating that the crisis that families are in, mentioned by some interviewees as a hindrance to meaningful informed consent, is seen to subside during the first weeks. Hence, further educational training may help address potential gaps in knowledge. This corresponds well with our previous findings in which we asked parents about their perspectives on participation in WGS research (Byrjalsen et al., 2018), and found varying views concerning the right time for approach, although the vast majority of interviewed parents had found an early approach acceptable (<4 weeks from diagnosis). However, as pointed out by a doctor in a study by Ardern‐Jones, Kenen, and Eeles (2005) of genetic testing at the time of cancer diagnosis, “nobody knows” what the right time to approach families is.

Smaller differences were seen between doctors (including pediatric oncologists and clinical geneticists) and nurses in our questionnaire. Doctors tended to approve of an earlier approach compared to nurses which might reflect the different duties of nurses and doctors: doctors are highly focused on treatment of the child with cancer (and, in our case, the potential for treatment adaptation based on WGS results), whereas nurses often take the role of general caretaker of the whole family.

### 4.3 Utility

Within the STAGING project, families are informed that inclusion will not alter the course of their child's treatment but may—if a cancer predisposition syndrome is found—result in different forms of surveillance posttreatment. The nature of surveillance is controversial as some pediatric oncologists are concerned by its implications, which led to clashes between those critical of surveillance and proponents within the relatively small medical specialty of pediatric oncology in Denmark when STAGING was implemented. McCullough et al. found that neither doctors nor parents found whole exome sequencing to be an ethically disruptive technology when applied to pediatric cancer patients with solid tumors, and that parents overwhelmingly welcomed the additional knowledge in their decision‐making (McCullough et al., 2016). On the other hand, Grove, Wolpert, Cho, Lee, and Ormond, (2014) found numerous differences in opinion within healthcare professionals views, as participants disagreed on who would be responsible for sequencing results (laboratory, patient, or healthcare provider) regarding what to disclose and at what indication, whether healthcare providers had sufficient knowledge to get informed consent from patients and deliver sequencing results. In our study, germline WGS emerged as a partially disruptive technology in the pediatric oncology clinic with differing practitioner views on its utility and relevance. History has shown how other so called “ethically disruptive” technologies have become mainstream in the clinic decades later (e.g., In vitro fertilisation). Yet, even if these practices have become mainstream, they still raise difficult ethical questions. The subject of much of modern medical ethics' most difficult questions are the flip side of all the good that comes from new technologies. The same will likely apply to germline WGS.

Roughly 100 families have received their results from WGS through the STAGING project, including both patients with pathogenic findings and patients without. Ongoing follow‐up qualitative studies will help us gain further knowledge into postreporting family perspectives on participation as well as on genetic testing in the family and potential lifelong surveillance.

### 4.4 Strengths and weaknesses

A weakness of this study concerns the study set‐up, in which the primary investigators AB and AW, also work within the STAGING study. And although AW is exactly engaged to address these issues, and regardless of anonymity, there is a risk that interviewees may have held some concerns back. Another weakness of the study concerns the questionnaire survey which was developed due to concerns raised by clinicians. Thus, the questionnaire was not validated through implementation in a pilot cohort prior to use in this study. Additionally, due to the convenience sampling used in this study, there is a risk that interviewees and respondents held a more positive view of the investigators and/or the STAGING study.

The strengths of this study include the combination of the investigators' experiences from their work on the ward, which included numerous contacts with clinicians, nurses, patients, and parents (when approaching families about STAGING), in‐depth interviews with—and survey data from—healthcare professionals. In addition, the scale of this study is a strength as key persons from all of Denmark's Pediatric Oncology Departments and Clinical Genetics Departments involved in the study at the time, were interviewed and received a questionnaire.

## 5 CONCLUSIONS

Healthcare professionals within pediatric oncology and genetics in Denmark are aware of the dilemmas caused by the intertwinement of clinical work and research. Informed consent poses a challenge regarding germline WGS, as patients cannot be fully informed of all potential outcomes (primary or secondary). Overall, the majority of participating healthcare professionals support approaching families within the first 4 weeks from diagnosis. Interviewees advocate for further education of families participating in WGS research and call for continued training of themselves on the subject of WGS. Healthcare professionals are divided in their views on surveillance.

## CONFLICT OF INTEREST

The authors declare no conflict of interest.

## AUTHOR CONTRIBUTION

AB and AW conducted the field work and did the initial data analysis, wrote the first draft of the paper, and handled the review process AC and US commented on the draft. AB submitted the manuscript.

## Supporting information

Supplementary Material

## Data Availability

The data supporting the findings of this study are available within this paper.
