# Supplementary material for: Germline whole genome sequencing in pediatric oncology in Denmark—Practitioner perspectives
Source: Mol Genet Genomic Med. 2020 Jun 4;8(8):e1276. doi: 10.1002/mgg3.1276 (PMC7434747; doi:10.1002/mgg3.1276)
Supplement: Supplementary file 1 — Supplementary Material [file MGG3-8-e1276-s001.docx]

Text box: Questions semi-structured interview guide

1. Why is it important to carry out as much research as you do on the pediatric oncology ward?

2. What do you need to be mindful of when you approach families about participation in a research project?

3. What do you see as some of the specific or important ethical issues that whole genome sequencing gives rise to for families?

4. In which ways do you envisage genetic information will be relevant for you as a practitioner?
